# Inguinal Hernia Repair in Pediatric Surgery Subspecialty Training: Safety and Clinical Outcomes of Open and Laparoscopic Surgery Performed by Trainees

**DOI:** 10.7759/cureus.105630

**Published:** 2026-03-21

**Authors:** Shohei Yoshimura, Emi Tsuji, Kengo Hattori, Jiro Tsugawa

**Affiliations:** 1 Department of Pediatric Surgery, Takatsuki General Hospital, Takatsuki, JPN

**Keywords:** laparoscopic inguinal hernia repair, open inguinal hernia repair, pediatric inguinal hernia, pediatric surgery subspecialty training, surgical education

## Abstract

Introduction

Inguinal hernia repair is essential in pediatric surgery training; however, few studies have examined the safety and outcomes of the procedure when performed by trainees. This study aimed to clarify the safety and clinical outcomes of open repair, laparoscopic percutaneous extraperitoneal closure (LPEC), and single-incision LPEC (SILPEC) for inguinal hernias in children, when performed by pediatric surgery trainees.

Methods

This single-center, retrospective cohort study included children aged <16 years who underwent conventional open repair, LPEC, or SILPEC for inguinal hernia between September 2015 and August 2023; patients who had comorbidities or underwent other procedures were excluded. Patient demographics, operative data, intraoperative and postoperative complications, and hernia recurrence were collected from medical records and analyzed.

Results

A total of 709 patients were eligible. Pediatric surgery trainees performed 325/372 (88%), 143/196 (73%), and 73/141 (52%) open, LPEC, and SILPEC procedures, respectively. Intraoperative complication rates (trainees vs. attending surgeons) were comparable between the open, LPEC, and SILPEC procedures (0% vs. 0%, p = 1.00; 0% vs. 0%, p = not available; 0% vs. 2%, p = 0.45, respectively). The postoperative complication rates (trainees vs. attending surgeons) were also similar between the open, LPEC, and SILPEC procedures (5% vs. 4%, p = 1.00; 6% vs. 8%, p = 0.86; 7% vs. 13%, p = 0.32, respectively). Hernia recurrence was not observed in any procedure.

Conclusion

Inguinal hernia repair performed by pediatric surgery trainees was not associated with increased intraoperative or postoperative complications. The clinical outcomes were favorable, with no hernia recurrence in open, LPEC, or SILPEC procedures, even when performed by pediatric surgery trainees.

## Introduction

Inguinal hernia repair is the most frequently performed procedure in pediatric surgery, making it an important component of surgical training for pediatric surgery trainees. However, pediatric surgeons must maintain surgical quality, ensure safety, and minimize complications irrespective of trainee involvement.

Inguinal hernia repair includes open and laparoscopic approaches, each requiring a range of fundamental surgical skills, including grasping, dissection, tissue retraction, ligation, port placement, and forceps handling. Currently, three procedures are predominant in Japan: conventional open repair, represented by the Potts procedure [1]; laparoscopic percutaneous extraperitoneal closure (LPEC); and single-incision LPEC (SILPEC). LPEC is a novel procedure for pediatric inguinal hernia, reported by Takehara et al. in 2006 [2]. SILPEC, which uses the LPEC technique and is limited to umbilical wounds, was developed by Uchida et al. in 2010 [3]. Recently, LPEC and SILPEC have gained widespread adoption for laparoscopic inguinal hernia repair in children across Japan and other Asian countries, replacing the conventional open approach and gaining popularity in recent decades [4].

Regarding surgical training, a limited number of studies have reported the safety and clinical outcomes of open or laparoscopic inguinal hernia repair performed by surgical trainees [5,6]. For instance, recurrence rates have been reported inconsistently based on the involvement of trainees and surgical experience. Yoshimura et al. suggested that there is no difference in recurrence rates between attending surgeons and surgical residents in open procedures [5], while Wilkiemeyer et al. indicated that junior residents have higher recurrence rates than senior residents in open procedures, but not in laparoscopic procedures [6]. This study aimed to clarify the safety and clinical outcomes of open repair, LPEC, and SILPEC for inguinal hernias in children, with procedures performed by pediatric surgery trainees.

## Materials and methods

Patient collection

All children under 16 years of age who underwent open repair, LPEC, or SILPEC for inguinal hernia between September 2015 and August 2023 at Takatsuki General Hospital, Takatsuki, Japan, were included. Patients with comorbidities related to other procedures were excluded. Patient demographics, operative data, intraoperative and postoperative complications, hernia recurrence, contralateral metachronous inguinal hernia (CMIH), and postoperative ascending testes were retrospectively collected from medical records. Postoperative complications were defined as those occurring within 30 days of surgery and classified according to the Clavien-Dindo classification [7]. Regular follow-up beyond 30 days postoperatively was not performed; therefore, hernia recurrence, CMIH, and postoperative ascending testis were diagnosed when patients presented to the outpatient clinic with symptoms.

Surgical procedures

Open repair was performed following either the Potts procedure [1] or the Lucas-Championnière procedure, depending on surgeon preference. Laparoscopic surgery was performed using LPEC or SILPEC, according to the reports of Takehara et al. [2] and Uchida et al. [3]. Prophylactic closure of the contralateral processus vaginalis was performed when observed during laparoscopic surgery. At our institution, LPEC was predominantly selected for boys, whereas SILPEC was preferred for girls. LPEC was performed in challenging scenarios for girls, such as patients weighing <5 kg and presenting with sliding hernias, considering the technical skills of the operator.

Operators and assistants

Each surgery was performed by pediatric surgery trainees or attending surgeons. Attending surgeons were defined as board-certified pediatric surgeons by the Japanese Society of Pediatric Surgeons, and pediatric surgery trainees were defined as individuals yet to obtain board certification in pediatric surgery. For pediatric surgery trainees, years of training in pediatric surgery were surveyed. The attending surgeon determined whether a trainee would perform the surgery as an operator prior to the procedure, based on the trainee’s skill level and the patient’s background. When trainees performed the surgery, the attending surgeon participated as an assistant and assumed responsibility for supervision.

Statistical analyses

Patient demographics, operative time, intraoperative and postoperative complications, and clinical outcomes such as recurrence, CMIH, and ascending testes were compared between trainees and attending surgeons, depending on the operator for each procedure. Continuous data are presented as mean ± standard deviation and were compared using the Student’s t-test. Categorical data are presented as percentages and were compared using Fisher’s exact test. Missing data were excluded from the statistical analyses. JMP® 15 (SAS Institute Inc., Cary, NC, USA) was used for statistical analyses. A p-value < 0.05 was considered statistically significant.

Ethical approval

This study was approved by the Institutional Review Board of Takatsuki General Hospital (approval no. 2023-39).

## Results

Patient collection

In total, 741 patients under 16 years of age underwent inguinal hernia repair at our institution between September 2015 and August 2023. Among these patients, 32 were excluded from this study because of comorbidities with other procedures, and 709 patients were included. Of these, open surgery was performed in 372 patients, with 325 (88%) performed by 12 pediatric surgery trainees (years of training: 0-4 years). LPEC and SILPEC were performed in 143/196 (73%) and 73/141 (52%) cases by seven (0-3 years of training) and five trainees (0-2 years of training), respectively. Open surgery, LPEC, and SILPEC involved six, five, and five attending surgeons, respectively; all attending surgeons had more than five years of experience in pediatric surgery.

Patient demographics

Patient demographics are presented in Table 1. LPEC was predominantly performed in boys, and SILPEC was performed in girls, resulting in fewer patients with hydrocele than in the open and LPEC procedures. Age and body weight at the time of surgery were similar between trainees and attending surgeons for all procedures. Most patients underwent day surgery in all procedures.

**Table 1:** Patient demographics ^†^Chi-square test, ^‡^t-test ASA-PS, American Society of Anesthesiologists physical status; LPEC, laparoscopic percutaneous extraperitoneal closure; SILPEC, single-incision laparoscopic percutaneous extraperitoneal closure

	Open (n = 372)				LPEC (n= 196)				SILPEC (n = 141)			
	Trainee (n = 326)	Attending (n = 46)	t-value/Chi-square value	p-value	Trainee (n = 143)	Attending (n = 53)	t-value/Chi-square value	p-value	Trainee (n = 73)	Attending (n = 68)	t-value/Chi-square value	p-value
Male (%)^†^	227 (70)	34 (74)	0.17	0.67	114 (80)	49 (93)	3.61	0.06	9 (12)	13 (19)	0.77	0.38
Age (years)^‡^	3.7 ± 2.8	3.6 ± 2.7	0.12	0.90	4.0 ± 2.8	3.3 ± 2.8	1.69	0.09	5.4 ± 3.6	4.5 ± 2.9	1.53	0.13
Body weight (kg)^‡^	14.4 ± 7.0	14.6 ± 7.6	-0.23	0.82	15.5 ± 6.9	14.1 ± 7.5	1.19	0.24	19.0 ± 10.4	16.3 ± 7.7	1.70	0.09
Preoperative diagnosis^†^			2.16	0.34			0.06	0.97			1.11	0.58
Right (%)	181 (56)	22 (48)	-	-	81 (57)	29 (55)	-	-	41 (56)	33 (49)	-	-
Left (%)	121 (37)	22 (48)	-	-	52 (37)	20 (38)	-	-	28 (38)	29 (43)	-	-
Bilateral (%)	24 (7)	2 (4)	-	-	10 (7)	4 (8)	-	-	4 (6)	6 (9)	-	-
Postoperative diagnosis^†^			2.16	0.34			3.68	0.16			3.93	0.14
Right (%)	181 (56)	22 (48)	-	-	45 (32)	10 (19)	-	-	18 (25)	11 (16)	-	-
Left (%)	121 (37)	22 (48)	-	-	24 (17)	8 (15)	-	-	8 (11)	15 (22)	-	-
Bilateral (%)	24 (7)	2 (4)	-	-	74 (52)	35 (66)	-	-	47 (64)	42 (62)	-	-
ASA-PS^†^			0.94	0.62			0.39	0.82			0.95	0.62
1 (%)	284 (87)	38 (83)	-	-	136 (95)	51 (96)	-	-	68 (93)	64 (94)	-	-
2 (%)	34 (10)	7 (15)	-	-	6 (4)	2 (4)	-	-	4 (6)	4 (6)	-	-
3 (%)	8 (2)	1 (2)	-	-	1 (1)	0 (0)	-	-	1 (1)	0 (0)	-	-
Day surgery (%)^†^	239 (73)	31 (67)	0.44	0.51	122 (85)	43 (81)	0.24	0.62	66 (90)	54 (79)	2.55	0.11
Hydrocele (%)^†^	112 (34)	14 (30)	0.12	0.72	53 (37)	17 (32)	0.23	0.63	7 (10)	2 (3)	1.61	0.20
Siding hernia (%)^†^	10 (10)	2 (17)	0.040	0.84	5 (4)	1 (2)	0.00	1.00	1 (2)	0 (0)	0.00	1.00
History of incarceration (%)^†^	19 (6)	3 (7)	0.00	0.74	10 (7)	0 (0)	2.59	0.11	2 (3)	5 (7)	0.76	0.38

Operative time analysis

In conventional open repair, the operative and anesthesia times were comparable for boys and girls, regardless of whether the operator was a trainee. Similarly, the operative, anesthesia, and pneumoperitoneum times in LPEC were comparable between trainees and attending surgeons in both boys and girls. However, in SILPEC for boys, operative, anesthesia, and pneumoperitoneum times were significantly longer for trainees than for attending surgeons (74.9 ± 22.3 vs. 48.1 ± 11.5 min, p < 0.01; 102.1 ± 22.8 vs. 77.2 ± 7.8 min, p < 0.01; 50.7 ± 22.2 vs. 27.6 ± 10.7 min, p < 0.01) (Table 2). In contrast, operative, anesthesia, and pneumoperitoneum times in SILPEC for girls did not show significant differences between trainees and attending surgeons, similar to open repair and LPEC (43.3 ± 17.5 vs. 40.7 ± 17.0 min, p = 0.41; 71.2 ± 18.2 vs. 71.0 ± 18.4 min, p = 0.94; 27.6 ± 13.2 vs. 26.7 ± 13.6 min, p = 0.72).

**Table 2:** Operative and anesthesia time ^‡^t-test, ^**^p < 0.01 LPEC, laparoscopic percutaneous extraperitoneal closure; NA, not available; SILPEC, single-incision laparoscopic percutaneous extraperitoneal closure

	Open (n = 372)				LPEC (n = 196)				SILPEC (n = 141)			
	Trainee (n = 326)	Attending (n = 46)	t-value	p-value	Trainee (n = 143)	Attending (n = 53)	t-value	p-value	Trainee (n = 73)	Attending (n = 68)	t-value	p-value
Male (n = 446)												
Operative time (minutes)^‡^	36.9 ± 15.8	39.3 ± 20.8	-0.79	0.43	41.1 ± 12.9	38.7 ± 12.7	1.08	0.28	74.9 ± 22.3	48.1 ± 11.5	3.71	<0.01^**^
Anesthesia time (minutes)^‡^	60.4 ± 18.3	63.7 ± 22.5	-0.94	0.35	69.3 ± 13.7	68.5 ± 14.0	0.31	0.76	102.1 ± 22.8	77.2 ± 7.8	3.68	<0.01^**^
Pneumoperitoneum time (minutes)^‡^	NA	NA	NA	NA	30.4 ± 12.5	31.1 ± 11.3	-0.34	0.73	50.7 ± 22.2	27.6 ± 10.7	3.25	<0.01^**^
Female (n = 263)												
Operative time (minutes)^‡^	32.3 ± 12.9	30.3 ± 15.6	0.52	0.61	38.4 ± 16.2	42.5 ± 9.6	-0.49	0.63	43.3 ± 17.5	40.7 ± 17.0	0.82	0.41
Anesthesia time (minutes)^‡^	54.7 ± 14.1	53.8 ± 16.9	0.22	0.83	67.8 ± 17.2	71.0 ± 7.8	-0.37	0.72	71.2 ± 18.2	71.0 ± 18.4	0.08	0.94
Pneumoperitoneum time (minutes)^‡^	NA	NA	NA	NA	23.4 ± 9.9	29.8 ± 9.5	-1.21	0.24	27.6 ± 13.2	26.7 ± 13.6	0.37	0.72

Safety of surgery performed by trainees

Only two patients had intraoperative complications, such as bowel injury and forceps failure (Table 3). No significant differences were observed in intraoperative complication rates between trainees and attending surgeons in open repair, LPEC, and SILPEC (0% vs. 0%, p = 1.00; 0% vs. 0%, p = not available; 0% vs. 2%, p = 0.58).

**Table 3:** Intraoperative and postoperative complications ^†^Chi-square test CMIH, contralateral metachronous inguinal hernia; LPEC, laparoscopic percutaneous extraperitoneal closure; NA, not available; PONV, postoperative nausea and vomiting; SILPEC, single-incision laparoscopic percutaneous extraperitoneal closure; SSI, surgical site infection

	Open (n = 372)				LPEC (n = 196)				SILPEC (n = 141)			
	Trainee (n = 326)	Attending (n = 46)	Chi-square value	p-value	Trainee (n = 143)	Attending (n = 53)	Chi-square value	p-value	Trainee (n = 73)	Attending (n = 68)	Chi-square value	p-value
Intraoperative complication (%)^†^	1 (0)	0 (0)	0.00	1.00	0 (0)	0 (0)	NA	NA	0 (0)	1 (2)	0.58	0.45
Other organ injury (%)	1 (0)	0 (0)	-	-	0 (0)	0 (0)	-	-	0 (0)	0 (0)	-	-
Forceps failure (%)	0 (0)	0 (0)	-	-	0 (0)	0 (0)	-	-	0 (0)	1 (2)	-	-
Postoperative complication (%) ^†^	15 (5)	2 (4)	0.00	1.00	8 (6)	4 (8)	0.03	0.86	5 (7)	9 (13)	0.97	0.32
SSI (%)	0 (0)	0 (0)	-	-	5 (4)	1 (2)	-	-	4 (6)	2 (3)	-	-
Stitch abscess (%)	3 (1)	0 (0)	-	-	2 (1)	3 (6)	-	-	0 (0)	3 (4)	-	-
PONV (%)	5 (2)	1 (2)	-	-	0 (0)	0 (0)	-	-	0 (0)	2 (3)	-	-
Bleeding/Hematoma (%)	2 (1)	0 (0)	-	-	0 (0)	0 (0)	-	-	0 (0)	0 (0)	-	-
Others (%)	5 (2)	1 (2)	-	-	1 (1)	0 (0)	-	-	1 (1)	2 (3)	-	-
Clavien-Dindo classification (Grade) ^†^			0.14	0.93			0.26	0.88			3.67	0.30
Ⅰ (%)	14 (4)	2 (4)	-	-	6 (4)	3 (6)	-	-	2 (3)	6 (9)	-	-
Ⅱ (%)	1 (0)	0 (0)	-	-	2 (1)	1 (2)	-	-	2 (3)	3 (4)	-	-
Ⅲ (%)	0 (0)	0 (0)	-	-	0 (0)	0 (0)	-	-	1 (1)	0 (0)	-	-
Recurrence (%) ^†^	0 (0)	0 (0)	NA	NA	0 (0)	0 (0)	NA	NA	0 (0)	0 (0)	NA	NA
CMIH (%) ^†^	15 (5)	2 (5)	0.00	1.00	0 (0)	0 (0)	NA	NA	0 (0)	1 (4)	0.00	0.97
Ascending testis (%) ^†^	3 (1)	0 (0)	0.00	1.00	0 (0)	0 (0)	NA	NA	0 (0)	0 (0)	NA	NA

The overall postoperative complication rate was higher for LPEC and SILPEC than for open repair because these laparoscopic procedures had a higher incidence of surgical site infection and stitch abscesses than in open repair. Postoperative complication rates (trainees vs. attending surgeons) were comparable between open repair, LPEC, and SILPEC procedures (5% vs. 4%, p = 1.00; 6% vs. 8%, p = 0.86; 7% vs. 13%, p = 0.32).

Clinical outcomes of surgery performed by trainees

No hernia recurrence was observed during any procedure (Table 3). Open repair of CMIH was performed in 17 (5%) patients and SILPEC in one (1%); all patients required either open or laparoscopic inguinal hernia repair for CMIH. No significant difference was observed in CMIH incidence between surgeries performed by trainees and those performed by attending surgeons (5% vs. 5% for open repair, p = 1.00; 0% vs. 4% for SILPEC, p = 0.97). Postoperative ascending testes were observed in only three (1%) patients who underwent open repair; however, no patient required orchidopexy for the ascending testes. The incidence of ascending testes was similar between cases managed by trainees and attending surgeons (1% vs. 0%, p = 1.00).

## Discussion

Inguinal hernia repair is the first stage in pediatric surgery subspecialty training, and most trainees undertake this procedure early in their training. At our institution, trainees under the supervision of an attending surgeon have performed 88% of conventional open repairs, 73% of LPEC, and 52% of SILPEC. Open repairs had been performed more frequently by trainees than LPEC and SILPEC because trainees received more assistance from attending surgeons during open repairs than during laparoscopic surgery.

Few studies have investigated the safety and clinical outcomes of inguinal hernia repair during residency training [5,6]. Our study clarified that pediatric surgery trainees safely performed both open and laparoscopic inguinal hernia repair in children, without increased intraoperative and postoperative complications. Furthermore, clinical outcomes were favorable in cases managed by trainees, with no instances of hernia recurrence observed. A previous study showed that the operative time for open inguinal hernia repair in children was longer when performed by trainees [5]. However, complications and hernia recurrence did not increase, even in cases managed by trainees [5]. Another report on laparoscopic inguinal hernia repair compared only the learning curve between trainees and attending surgeons, without investigating the safety and clinical outcomes [8]. In our analysis of operative times, only SILPEC for boys required a longer operative time in trainee cases. The operative time remained comparable between trainees and attending surgeons for open repair, LPEC, and SILPEC for girls. SILPEC is a unique procedure that necessitates the smooth handling of the SILPEC forceps. Particularly in boys, dissection around the testicular vessels and vas deferens, with the SILPEC forceps, may present challenges for inexperienced trainees.

Both open and laparoscopic procedures require a range of fundamental skills in pediatric surgery, making these procedures important for surgical training. However, a major problem worldwide has been the decreasing opportunities for surgical training in recent years [9]. The total number of pediatric operative cases, including inguinal hernia repair during residency training, has decreased in the United States of America during the past two decades [10], and the situation is similar in Japan. As the surgical volume of pediatric inguinal hernias has been identified as a risk factor for hernia recurrence [11], it becomes imperative to contemplate how to sustain efficient surgical training while maintaining favorable clinical outcomes.

This study had a few limitations. First, this was a retrospective study conducted at a single institution, with a limited number of patients. Although a prospective study is not recommended, a larger retrospective study using a national database should be conducted in future studies. Second, selection bias may have occurred, as challenging cases were consistently managed by attending surgeons rather than by trainees. Third, an incomplete postoperative follow-up might underestimate the rate of postoperative complications, including inguinal hernia recurrence. Finally, evaluating the extent of the attending surgeons’ assistance to trainees during surgery presented a challenge.

## Conclusions

Inguinal hernia repairs performed by pediatric surgery trainees were not associated with increased intraoperative and postoperative complications in the open, LPEC, and SILPEC procedures. No statistically significant differences in complications were observed; however, these findings should be interpreted with caution, given the retrospective design and potential selection bias.
